# Numerical Model for Magnetic Fluid Hyperthermia in a Realistic Breast Phantom: Calorimetric Calibration and Treatment Planning

**DOI:** 10.3390/ijms20184644

**Published:** 2019-09-19

**Authors:** Arkadiusz Miaskowski, Mahendran Subramanian

**Affiliations:** 1 Department of Applied Mathematics and Computer Science, University of Life Sciences Lublin, 20-950 Lublin, Poland; arek.miaskowski@up.lublin.pl; 2 Department of Bioengineering and Department of Computing, Royal School of Mines, Imperial College London, London SW7 2AZ, UK

**Keywords:** magnetic fluid hyperthermia, breast cancer, treatment planning, numerical modelling

## Abstract

This paper aims to apply a proposed, based on calorimetric measurements, a reliable numerical model for magnetic fluid hyperthermia (MFH) treatment planning of breast cancer. Furthermore, we perform a comparative analysis of magnetic nanoparticles (MNPs) and tumour tissue interactions by means of the magnetic-field-dependent Néel and Brownian relaxation times. The analysis was based on an anatomically correct breast model (developed in-house) and a modified linear response theory, which was applied to investigate the heat dissipation from the magnetic nanoparticles dispersed in the breast tumour. The calculations of the single-domain magnetic power losses were conducted for a case where the magnetic field value and the applied frequency were known, but also for the different concentrations of the MNPs in the tumour. Two scenarios were considered: The MNPs mobilised and immobilised in the tumour. In parallel, the eddy currents effect, together with the related temperature distributions, were considered in order to analyse safety issues. By changing the MNP concentration in the tumour, the corresponding temperature distributions were calculated. The eddy current effect, together with the related temperature distribution, were considered in order to analyse safety issues. Varying the MNP concentration in the tumour, the corresponding temperature distribution was calculated. Moreover, the cumulative equivalent minutes at 43 ℃ were analysed. In the anatomically correct breast phantoms, the tissue location can lead to “hot spots” due to the eddy current effect and subsequently to the high gradients of the temperature. That is why the analysis of safety issues related to the overheating side effect should be taken into consideration during the treatment planning of magnetic fluid hyperthermia. The phenomenon of heat dissipation from MNPs is very sophisticated and depends on their concentration, the distribution and the relaxation mechanism in the tumour, together with magnetic field strength and frequency. Furthermore, we inferred that the phenomenon of heat dissipation from MNPs equally depends on MNP-tissue interactions, and it can lead to 30% differences in the power assessment. Nevertheless, the aforementioned factors should be considered in parallel using anatomical, volume-dependent models to enhance the efficiency of in vivo treatment.

## 1. Introduction

Novel therapy methods without negative impacts are needed to reduce mortality rates in patients with breast cancer. One such method is magnetic fluid hyperthermia (MFH) in which magnetic nanoparticles (MNPs) dissipate heat during radiofrequency (RF) application [1,2,3,4,5], activating biochemical pathways leading to necrosis or apoptosis [6,7,8,9]. In the past decade, attention has been given to MFH treatment for breast cancer either on its own or in combination with chemotherapy and radiotherapy [10,11,12,13,14]. However, dexterous techniques of modelling and validation will help to improve and standardise this potential clinical treatment. Effective MFH is accomplished when maximum cytotoxicity with low or no side effects are attained with a limited dosage of Alternating Magnetic Field (AMF) susceptible MNPs, so suitable heat characterisation of MNPs and treatment planning should be carried out prior to therapy. Besides, ethical factors, cost-effective clinical trials and infrastructure requirements restrict the number of teams performing in vivo studies, hampering significant progress in understanding MFH for the therapy of breast cancer. Furthermore, constraints such as those for the pain threshold of 4.85 × 108 A/m/s for induced eddy currents in patients during MFH treatment [9] and the field limit being sufficient to saturate all the particles within the distribution at a particular frequency, limit MFH treatment. However, it is possible to use virtual breast phantom models to evaluate the efficacy of MFH and the risk assessment of the radiofrequency.

Three dimensional (3-D) models were used in the past to investigate the effects of MNP volume fraction, nanocomposite geometry, and treatment parameters on thermal profiles regarding female breast cancer [15,16,17]. However, the proposed models were simplified to the semi-ellipsoidal block, the semi-sphere or to the anatomically correct shape without naturalistic breast tissues. This study aims to develop an improved numerical breast phantom model derived from human magnetic resonance images [18] to obtain a lifelike model. Moreover, the literature lacks numerical models, which include 3-D temperature distribution due to eddy currents and single-domain magnetic power losses in anatomically correct phantoms. Furthermore, the numerical investigation conducted in this work was based on the results received from calorimetric measurements of the ferrofluid using the magneTherm^TM^ system [19,20], i.e., the numerical model developed for heat generation terms. Specific loss powers (SLP) were validated with calorimetric measurements and are now used to analyse breast cancer hyperthermia treatment mediated by magnetic fluid.

It is clear that the reported SLPs values [20] do not reflect the situation one might expect to find in human tissues where, for example, blood perfusion, spatial tissues location and magnetic nanoparticles–tissue interactions affect heat dissipation. That is why, in this work, the reported SLPs values together with the numerical model, have been transposed to realistic breast cancer.

Our previous studies presented mathematical models, i.e., the single-domain magnetic power losses model and the eddy current model. Both of them were used later in the modified Pennes equation to evaluate a temperature distribution. The current work details the anatomically correct breast model used in the simulations and its parameters, followed by several analyses of volumetric power losses, which determine spatial temperature distribution as a function of time for various concentrations of magnetic fluid preparations. Furthermore, mobilised and immobilised MNPs in the tumour were considered.

## 2. Results and Discussion

The mathematical model described in the previous section was used to investigate the temperature distribution during MFH. Different magnetic fluid concentrations were applied in the tumour to control the temperature in the target tissue, then the tumour position in the breast model was determined by maximum electrical loss. These power density values are referred to as “hot spots”. In addition to the power losses due to the eddy current effect, a power dissipation from magnetic nanoparticles should be considered in parallel when dealing with MFH treatment planning. Moreover, particular interest was paid to the comparative analysis of MNPs–tissue interactions, as in MFH, and the heating rates of MNPs which were determined using Néel and Brownian losses, which can be inappropriate for MFH treatment. For a reliable model of heating, two cases were considered, i.e., the MNPs free to move in the tumour (Néel and Brownian relaxations included in the tumour) and the MNPs immobilised in the tumour (in which only Néel relaxation takes place).

### 2.1. Breast Phantom Model

**Figure 1:**
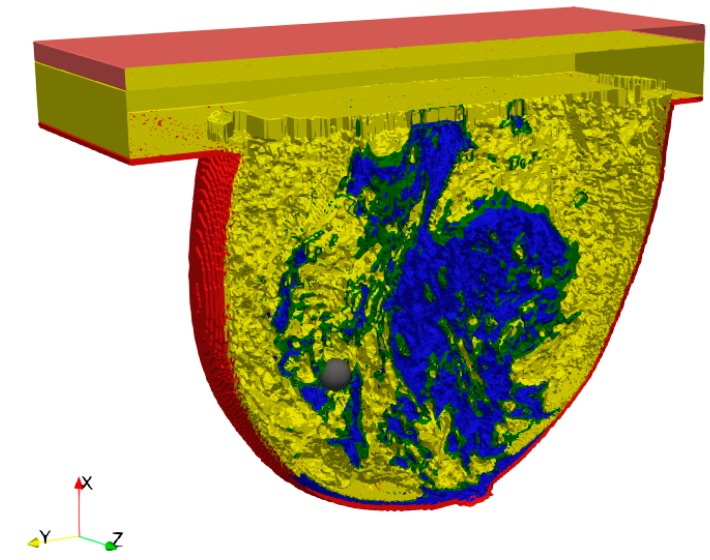
Breast phantom model—breast gland (blue), beast fat (yellow), fat (green), skin (red), muscle (orange) and tumour (grey).

In our previous study, the breast model containing only skin, breast fat and tumour tissues was evaluated based on field application by a pancake coil [17]. In the present study, more tissues were added to achieve a very detailed anatomical structure. The breast phantom model was taken from [18], and it was assigned to the Class 3 category—heterogeneously dense (51–75% glandular). The following breast tissues were included in the model: Breast gland (blue), breast fat (yellow), fat (green), skin (red) and muscle (orange) as shown in Figure 1. Moreover, the physical parameters of the tumour were considered as the same as the muscle tissues ones but with perfusion. The physical parameters of tissues were calculated for the frequency *f* = 171 kHz based on the Information Technologies in Society (IT’IS) database [21]. The parameters are shown in Table 1, where *HTR =* $\rho_{b}c_{b}\rho \omega$ is the heat transfer rate.

In our previous work [17], the tissues included in the breast phantom models were averaged due to their volume fraction together with their physical parameters. Such approximation can be useful while planning the treatment when the applicator position pattern is required. In the current work, the model was improved, and the effect of the eddy current and “hot spots” (the places where high values of power losses may occur due to the anatomical configuration of breast tissues) were examined. The model cross-section is shown in Figure 1.

### 2.2. Eddy Currents Effect on Temperature Distribution

The authors would like to emphasise that the aim of this part of the study is not the design a breast cancer applicator, but to fulfil the condition for the magnetic field strength ($H_{\max}=10.3\mathrm{kA}/m$ in the middle of the applicator) and the frequency, as was performed during the calorimetric experiments using the magneTherm^TM^ system. The location of the spherical tumour (2 mL volume) in the breast model was determined by the maximum volumetric eddy power density ($P_{\mathrm{eddy}}$) to consider the worst-case scenario.

**Figure 2:**
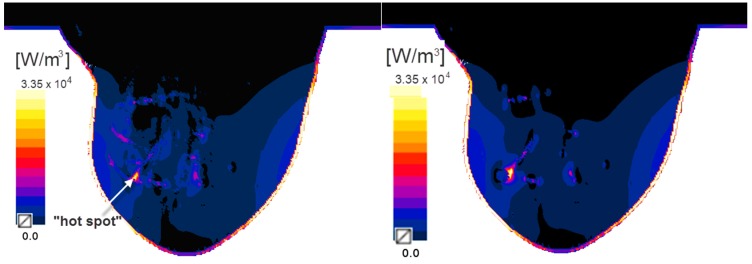
Distribution of the power density due to eddy currents in the breast model without the tumour (left) and with the tumour emended in the “hot spot” (right). The arrow (white) indicates the “hot spot”.

The volumetric power density due to the eddy currents was evaluated for frequency $\;f_{1}=171\;\mathrm{kHz}$, and is shown in Figure 2. It is obvious that the highest value of eddy power density occurred on the skin tissue, reaching 70 kW/m^3^. That is why the data was rescaled to the maximum value (35.5 kW/m^3^), i.e., to one of the “hot spots”, which were placed in the model excluding the skin layer to improve visualisation. It is important to note that the presence of “hot spots” should be considered during treatment planning as they are hard to predict and they can limit the amount of energy that can be deposited in a patient’s body. Moreover, one should remember that maximal electrical power losses will occur on the surface of the tumour as the eddy currents have to circulate around the tumour centre where they are the smallest. This sometimes can be used as an advantage because high-conductivity tumours surrounded by low-conductivity tissues will have a local eddy current flowing around the approximate centre of the tumour leading to increased heating of deep-seated tumours.

**Figure 3:**
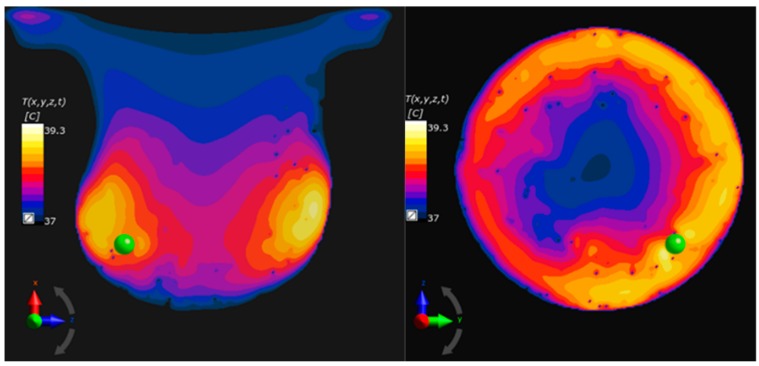
Temperature distribution (cross-section) in the breast model after 1800 s due to the eddy current effect together with the tumour position (xz- and zy-cross-section).

In our case, to investigate the worst-case scenario, the tumour was positioned in one of the “hot spots”, and the power density was calculated (see Figure 2—right). One can see that the tumour position has changed the power distribution pattern and the “hot spot” shifted due to the physical properties of the tumour. However, taking the temperature distribution into account (see Figure 3), the area where the temperature rises above 39 °C (which is relatively high), means that overheating of tissues can occur primarily on the skin.

### 2.3. Single-Domain Magnetic Power Loss Analysis

As the first step, the single-domain magnetic power losses for both the mobilised and the immobilised MNPs were calculated. The ferrofluid concentration was the same as what is was during the calorimetry experiments (φ=5.0 mg/mL), and it was used as a reference. The parameters which have been used in Formula (1) are provided in Table 2.

**Figure 4:**
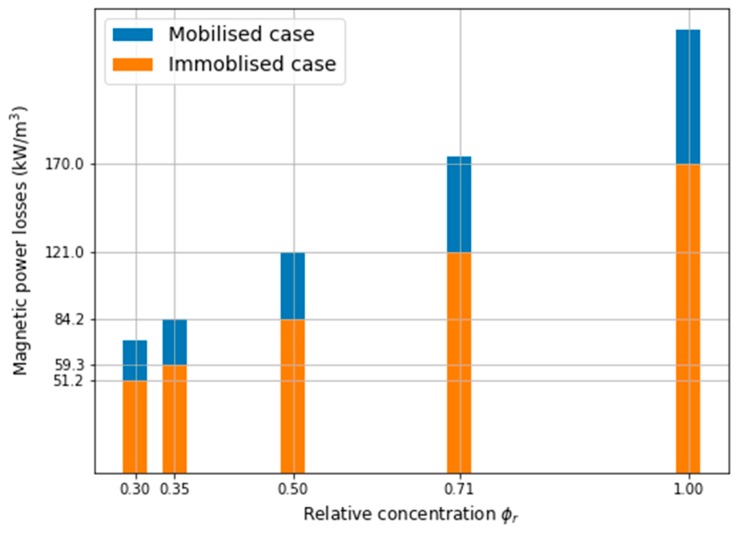
Single domain magnetic power losses—magnetic nanoparticles (MNPs) mobilised vs MNPs immobilised in the tumour.

Next, the ferrofluid concentration was changed $\left(\phi_{r}\right)$ to control the temperature in the tumour. The values of maximum magnetic power losses for the different relative concentrations $\phi_{r}$ are given in Table 3 and also presented in Figure 4.

Taking into account the single-domain magnetic power losses, one can see that the powers had decreased by 30% when MNPs were immobilised in the tumour. It is also easy to linearly approximate both cases as follows: $P_{\mathrm{mobilzed}}=244.4\phi_{r}-0.7826$, $P_{\mathrm{immoblized}}=170.3\phi_{r}-0.2539$ in order to predict the magnetic power losses for other magnetic fluid concentrations.

**Figure 5:**
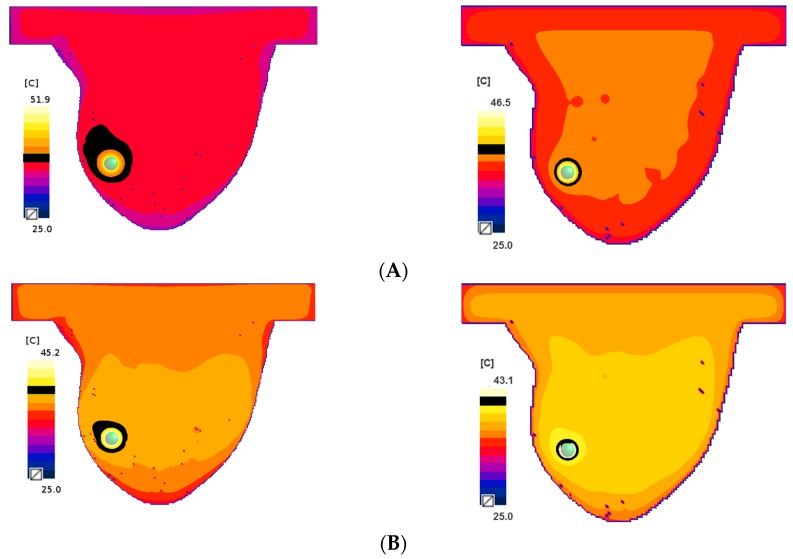

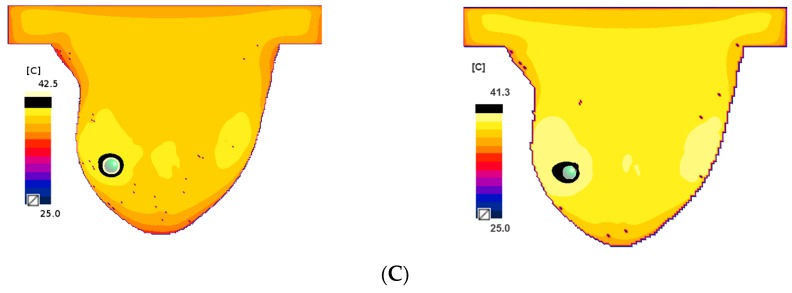
XY-cross section temperature distribution passing through the middle of the tumour after 1800 s for different concentrations: $\phi_{r}$ (**A**) $\;\phi_{r}=1.0$, (**B**) $\;\phi_{r}=0.5$ and (**C**) $\;\phi_{r}=0.35$. The mobilised MNPs are on the left, and the immobilised MNPs is on the right. The black colour indicates the 40 °C area.

**Figure 6:**
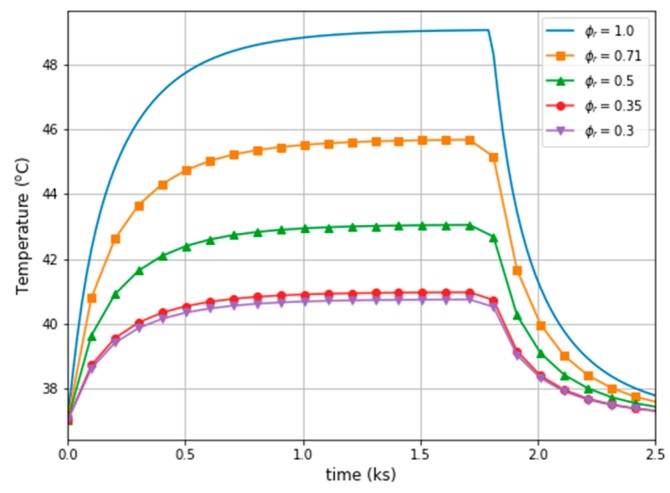
Temperatures over time in the middle of the tumour for different magnetic fluid concentrations (mobilised case).

For both cases (the mobilised MNPs on the left and the immobilised MNPs on the right) in Figure 5, the temperature distribution patterns are shown in the breast phantom for different concentrations of the ferrofluid after 1800 secs of heating. The black area in Figure 5A, 5B, 5C indicates the temperature of 40 °C for different $\phi_{r\;}$= [1, 0.5, 0.35]. Moreover, the temperatures over time in the middle of the tumour are presented in Figure 6 for the mobilised case.

**Figure 7:**
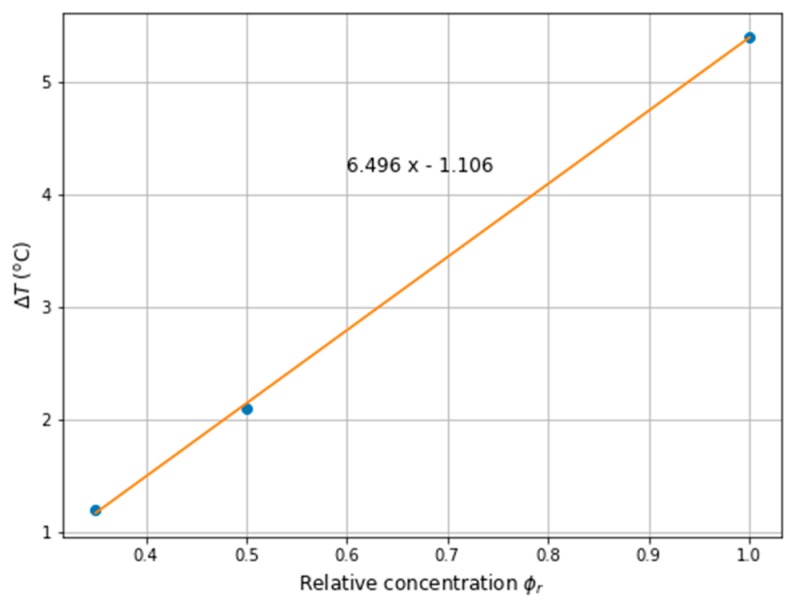
The temperature difference ΔT between mobilised and immobilised cases over ferrofluid relative concentration in the tumour together with linear approximation.

In the case of the immobilised MNPs in the tumour, it can be seen that both the maximum power dissipation (see Table 3), as well as the temperature, has reduced (see Figure 6). For the reference concentration, $\phi_{r}=1.0$, the power decreased by 30%, while the temperature difference, ΔT, was approximately 5.4 ℃ when comparing the two cases of MNPs in the tumour. Nevertheless, the relationship between MNPs concentration and the temperature increase is not straightforward, for example, for $\phi_{r}=0.5$ the temperature difference is ΔT=2.1 ℃ and for $\phi_{r}=0.35$ it is ΔT=1.2 ℃. However, in order to compare the aforementioned cases, the concentration, $\phi_{r}$, was changed for the corresponding values of the power dissipation (for example, to receive $P_{\mathrm{moblil}}=121.0\;\mathrm{kW}/m^{3}$, the concentration should be $\phi_{r}=0.5$ for the mobilised MNPs, while for immobilised MNPs concentration should be $\phi_{r}=0.71$, see Table 3). Finally, Figure 7 presents the relative concentration as the function of the temperature difference between the two cases together with its linear approximation.

**Figure 8:**
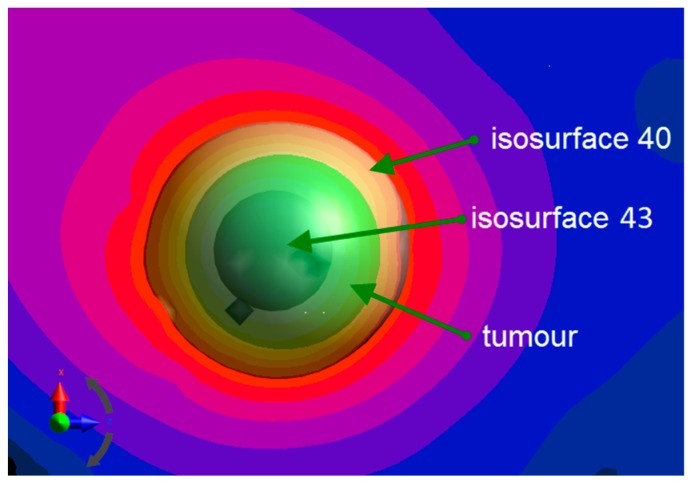
Isosurfaces 40 ℃ and 43 ℃ for concentration: $\phi_{r}=0.35$—mobilised. The tumour is green.

According to the data in Table 3 and Figure 5, it is possible to control the temperature in the tumour and its vicinity by changing the magnetic fluid concentration. The volumetric power density, required to raise the temperature to 43 ℃ in the tumour was at least $84.2\;\mathrm{kW}/m^{3}$, as depicted in Figure 8, where the isosurface 43 ℃ can be seen inside the tumour volume.

**Figure 9:**
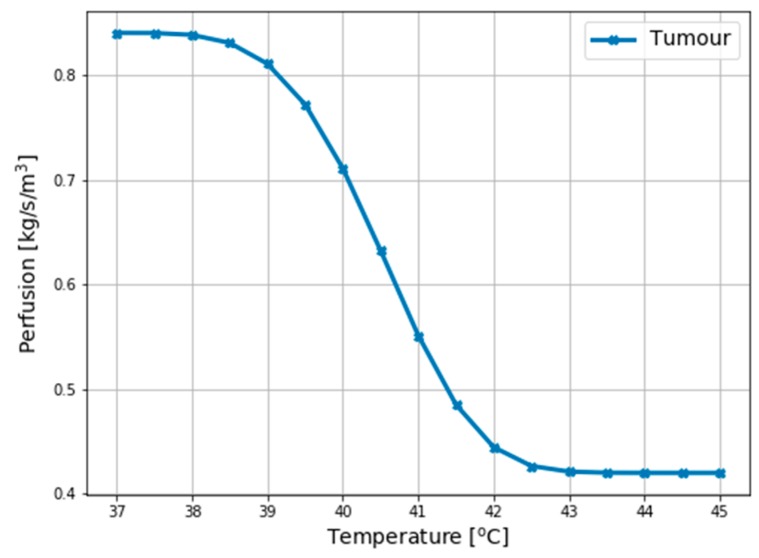
A nonlinear model of temperature-dependent blood perfusion for tumour.

However, in order to reach 43 ℃ in the whole tumour volume $121.0\;\mathrm{kW}/m^{3}$ was required but, in this case, the temperature inside the tumour was 45 ℃. On the other hand, it should be underlined that the heating power may have increased due to a possible agglomeration of MNPs in the tumour. Moreover, the heterogeneous temperature distribution in the tumour vicinity, which can be observed in Figure 4, Figure 6 and Figure 9 may lead to high-temperature gradients even if the eddy currents power losses are minimal when compared with the magnetic power ones. This phenomenon should be considered in patient treatment planning.

## 3. Materials and Methods

### 3.1. Single-Domain Magnetic Power Losses Model

When polydisperse magnetic nanoparticles in the superparamagnetic regime are exposed to an AMF with the given magnetic field strength, $H_{\max}$, and frequency, *f*, the magnetisation lags behind the external field. According to linear response theory (LRT), the heat dissipation in W/kg can be expressed as the specific loss power (SLP) [23]:

(1) $$SLP_{LRT}=\int_{0}^{V}{\pi \mu }_{0}\chi^{\prime \prime }H_{\max}^{2}f\rho V_{M}g\left(\mu ,s,V_{M}\right)dV\;\;\left(\frac{W}{\mathrm{kg}}\right)$$

where $g\left(\mu ,\;s,V_{M}\right)$ is the volume-weighted distribution (log-normal distribution), ρ and $V_{M}$ are the density and the magnetic volume of MNP, respectively, and $\chi^{\prime \prime }$ is the average out-of-phase component of the susceptibility given by:

(2) $$\chi^{\prime \prime }=\frac{\mu_{0}m}{3k_{B}T}\;\frac{2\pi f\tau }{\left(1+{\left(2\pi f\tau \right)}^{2}\right)}$$

where $k_{B}$ is the Boltzmann constant, $m=M_{s}V_{M}$ is the magnetic moment where $M_{s}$ is the magnetic saturation and τ is the Néel-Brown relaxation time. The Néel ($\tau_{N}$) and Brownian ($\tau_{B}$) metrics, as dependent on an external magnetic field (*H*_max_), are given by [23]:

(3) $$\begin{array}{c} {\left[\tau_{N}\left(H_{\max}\;\;\right)\right]}^{2}=f_{0}\left(1-h^{2}\right)\{\left(1+h\right)\exp\left[\left(-\frac{KV}{k_{B}T}\right){\left(1+h\right)}^{2}\right]+\\ +\left(1-h\right)\exp\left[\left(-\frac{KV}{k_{B}T}\right){\left(1-h\right)}^{2}\;\right]\} \end{array}$$

(4) $${\left[\tau_{B}\left(H_{\max}\right)\right]}^{-1}=\tau_{B}^{-1}{\left(1+0.07{\left(\frac{\mu_{0}\mu H_{\max}}{k_{B}T}\right)}^{2}\right)}^{0.5}$$

where $\tau_{B}=\frac{3\eta V_{H}}{k_{B}T}$ and η is the viscosity, $h=\frac{H_{\max}}{H_{k}}\;$ and $\mu_{0}H_{k}=\frac{2K}{M_{s}}$ represents the anisotropy field and $f_{0}=10^{9}\;s^{-1}$ is the attempt frequency. Finally, the existence of two relaxation times leads to the effective relaxation time given by $\tau {\left(H_{\max}\right)}^{-1}=\tau_{N}{\left(H_{\max}\right)}^{-1}+\tau_{B}{\left(H_{\max}\right)}^{-1}$. In this case, it is worth mentioning, that the above model contains few simplifications such as follows: All the MNPs have spherical shapes, MNPs do not create clusters or chains, and therefore there are not any inter-particle interactions and finally—the hydrodynamic particle size distribution is uniform. One should realise that if the concentration of the MNPs in the ferrofluid is relatively small and, for example, does not exceed 5 mg/mL, the effects caused by dipole–dipole interactions between the nanoparticles are considered insignificant at room temperature. Thus, in our case, the nanoparticles were considered to be magnetically isolated from each other.

### 3.2. Eddy Current Effect Model

When human tissues are exposed to an alternating electromagnetic field, the eddy current effect is observed due to non-zero conductivity of the tissues, which finally leads to their heating.

The magnetic field strength (H) generated by an applicator can be solved using the magnetic vector potential (A):

(5) $$-\nabla^{2}A=\mu_{0}J_{s}$$

where $J_{s}$ is the current density vector (A/m^2^) and $H=\mu_{0}^{-1}\nabla \times A$. In order to include a volumetric power density ($P_{\mathrm{eddy}}$) produced by an eddy current ($J_{\mathrm{eddy}}$) effect, a magneto-quasi static algorithm was applied. Considering the scalar potential (*u*) together with a magnetic vector potential (**A**), one can formulate the issue as:

(6) $$J_{eddy}=-\sigma \nabla u-\sigma \omega A$$

where σ is the conductivity (S/m) and ω = 2π*f* is the angular frequency. Finally, $P_{\mathrm{eddy}}$ can be expressed as:

(7) $$P_{\mathrm{eddy}}=\frac{J_{\mathrm{eddy}}^{2}}{\sigma }$$

Discussing the eddy current or Foucault current is essential due to their effect on different types of tissues when exposed to a time-varying magnetic field. The eddy current effect can lead to unwanted non-specific heating of healthy tissues, so it must be considered in the applicator design and during treatment planning.

### 3.3. Pennes Equation

A model dedicated to the numerical analysis of heat transfer in human tissues was proposed by Pennes [24]. In this model, blood perfusion is assumed to be uniform throughout the tissue and all heat leaving the artery is absorbed by the local tissue, with no venous rewarming. Moreover, the Pennes model is limited to the perfusion source term, i.e., the arterial temperature is assumed to be equal to the body core temperature.

To account for the strong temperature dependence due to bio-regulatory processes [25], the breast model tissues perfusion rates were assumed to be constant (ω(T)=const.) while the tumour perfusion was assumed nonlinear according to the following formula [26]:

(8) $$\omega_{\mathrm{tumour}}\left(T\right)=0.416+0.416\exp\left(-\frac{{\left(T-37\;℃\right)}^{4}}{220}\right)\;\;\;\;\left(\frac{\mathrm{kg}}{m^{3}s}\right)$$

which indicates the mass flow rate as presented in Figure 9.

Hence, the temperature distribution in the breast model was investigated using the modified bio-heat transfer equation:

(9) $$\rho c\frac{\partial T}{\partial t}=\nabla \cdot \left(\kappa \nabla T\right)+\rho_{b}c_{b}\rho \omega \left(T\right)\left(T_{b}-T\right)+\rho \mathrm{HGR}+\rho SLP_{\mathrm{LRT}}+P_{\mathrm{eddy}}\;\;\left(\frac{W}{m^{3}}\right)$$

where ρ is the tissue density, *c* is the tissue-specific heat, $\rho_{b}$ is the density of blood, $c_{b}$ is blood specific heat, κ is the tissue thermal conductivity, *ω*(T) is the perfusion rate, $T_{b}$ is the arterial blood temperature [C], *T* is the local temperature, HGR is the metabolic heat generated rate and $SLP_{\mathrm{LRT}}\;$ is the external power losses due to magnetic nanoparticles. The convection boundary condition (Robins type) was used:

(10) $$\kappa \frac{\partial T}{\partial n}=h\left(T_{\mathrm{ext}}-T\right)$$

where *h* is the heat transfer coefficient, and *κ* is the thermal conductivity of the skin layer.

## 4. Conclusions

The modified Pennes heat equation with Robin’s boundary condition to compute the temperature distribution within the realistic breast phantom was investigated. The single-domain magnetic and eddy current mediated power loss densities were applied as the parallel external heat sources. It was shown that due to the anatomical structure of the breast tissues, the “hot spots” could occur together with high-temperature gradients. That is why this phenomenon should be considered during treatment planning. 

More importantly, the distribution of the magnetic power losses along with the temperature was not homogeneous in the cancer area. It was also demonstrated that the viscosity played a crucial role, and the magnetic fluid concentration was critical for effective MFH treatment. The behaviour of MNPs within the tumour volume was also analysed as their lack of mobilisation leads to lower power dissipation. Consequently, it is vital to know if the MNPs are free to move in the interstitial fluid or whether they are immobilised in the tumour tissue. Thus, magnetic nanoparticles–tissue interactions hold the equal potential to affect treatment. 

To conclude, from the MFH-treatment planning point of view, it is crucial to prevent healthy surrounding tissues from overheating, so particular attention should be given to the temperature distribution in the tumour vicinity. The accuracy of magnetic fluid thermal therapy determines its role in prospective treatment planning for the individual patient because inadequate thermal doses to the tumour can cause a failure of treatment. Hence, the proposed numerical model will help to calibrate magnetic fluid temperature measurements when exposed to radiofrequency, and realistic models like the one presented here will enable in silico evaluation of MFH efficiency prior to clinical treatment.

## Figures and Tables

**Table 1:** Physical parameters of breast tissues for *f* = 171 kHz [21]. Muscle tissue parameters were set for the tumour.

Tissue	ρ	σ	*c*	κ	HGR*	HGR**
	[kg/m^3^]	[S/m]	[J/kg/C]	[W/m/C]	[W/kg]	[W/m^3^/C]
Skin	1109.0	0.17	3390.5	0.3722	1.647	7969.16
Muscle (tumour)	1090.4	0.355	3421.2	0.4949	0.9061	2705.95
Breast gland	1040.5	0.5412	2960.0	0.3345	2.323	10542.6
Fat	911.0	0.057	2348.3	0.21145	0.5	2012.82
Breast fat	911.0	0.0222	2348.33	0.209	0.7278	2892.22

HGR*—Heat Generation Rate, HTR**—Heat Transfer Rate.

**Table 2:** Data used to calculate single-domain magnetic power losses [20].

*H_max_*	K	k_B_	T	η	δ	Log-normal
kA/m	kJ/m^3^	J/K	K	kg/m/s	nm	*g*(µ, s, *V*_M)_
92.0	30.0	1.38^−23^	298.0	8.94^−4^	2.0	*g*(15.2, 0.19, *V*_M_)

**Table 3:** Single-domain (S-D) magnetic power losses with regard to the relative concentration ($\phi_{r}$) of ferrofluid in the tumour [22].

$\phi_{r}$	S-D Magnetic Power (Mobilised)	S-D Magnetic Power (Immobilised)
	kW/m^3^	kW/m^3^
1.0	243.0	170.0
0.71	174.0	121.0
0.5	121.0	84.2
0.35	84.2	59.3
0.3	72.8	51.2
